# In Vitro Rapid Antigen Test Performance with the SARS-CoV-2 Variants of Concern B.1.1.7 (Alpha), B.1.351 (Beta), P.1 (Gamma), and B.1.617.2 (Delta)

**DOI:** 10.3390/microorganisms9091967

**Published:** 2021-09-16

**Authors:** Sabrina Jungnick, Bernhard Hobmaier, Lena Mautner, Mona Hoyos, Maren Haase, Armin Baiker, Heidi Lahne, Ute Eberle, Clara Wimmer, Sabrina Hepner, Annika Sprenger, Carola Berger, Alexandra Dangel, Siegfried Ippisch, Sonja Hahner, Manfred Wildner, Bernhard Liebl, Nikolaus Ackermann, Andreas Sing, Volker Fingerle

**Affiliations:** 1 Public Health Microbiology Unit, Bavarian Health and Food Safety Authority, 85764 Oberschleißheim, Germany; sabrina.jungnick@lgl.bayern.de (S.J.); bernhard.hobmaier@lgl.bayern.de (B.H.); heidi.lahne@lgl.bayern.de (H.L.); ute.eberle@lgl.bayern.de (U.E.); clara.wimmer@lgl.bayern.de (C.W.); sabrina.hepner@lgl.bayern.de (S.H.); annika.sprenger@lgl.bayern.de (A.S.); carola.berger@lgl.bayern.de (C.B.); alexandra.dangel@lgl.bayern.de (A.D.); nikolaus.ackermann@lgl.bayern.de (N.A.); andreas.sing@lgl.bayern.de (A.S.); 2 Unit of Molecular Biologic Analytics and Biogenetics, Bavarian Health and Food Safety Authority, 85764 Oberschleißheim, Germany; lena.mautner@lgl.bayern.de (L.M.); mona.hoyos@lgl.bayern.de (M.H.); maren.haase@lgl.bayern.de (M.H.); armin.baiker@lgl.bayern.de (A.B.); 3 Bavarian Pandemic Warehouse, Bavarian Health and Food Safety Authority, 85764 Oberschleißheim, Germany; siegfried.ippisch@lgl.bayern.de; 4 Protein Biochemistry, Mikrogen GmbH, 82061 Neuried, Germany; hahner@mikrogen.de; 5 Walther Straub Institute of Pharmacology and Toxicology, Faculty of Medicine, Ludwig Maximilians-Universität, 80539 Munich, Germany; manfred.wildner@lgl.bayern.de (M.W.); bernhard.liebl@lgl.bayern.de (B.L.); 6 Bavarian State Institute of Health, 85764 Oberschleißheim, Germany

**Keywords:** SARS-CoV-2 rapid antigen tests, rapid antigen test performance, B.1.1.7 (Alpha), B.1.351 (Beta), P.1 (Gamma), B.1.617.2 (Delta), variants of concern (VOCs), human corona viruses (hCoVs), cross-reactivity

## Abstract

Rapid antigen tests (RATs) are an integral part of SARS-CoV-2 containment strategies. As emerging variants of concern (VOCs) displace the initially circulating strains, it is crucial that RATs do not fail to detect these new variants. In this study, four RATs for nasal swab testing were investigated using cultured strains of B.1.1 (non-VOC), B.1.1.7 (Alpha), B.1.351 (Beta), P.1 (Gamma), and B.1.617.2 (Delta). Based on dilution series in cell culture medium and pooled saliva, the limit of detection of these RATs was determined in a laboratory setting. Further investigations on cross-reactivity were conducted using recombinant N-protein from seasonal human coronaviruses (hCoVs). RATs evaluated showed an overall comparable performance with cultured strains of the non-VOC B.1.1 and the VOCs Alpha, Beta, Gamma, and Delta. No cross-reactivity was detected with recombinant N-protein of the hCoV strains HKU1, OC43, NL63, and 229E. A continuous evaluation of SARS-CoV-2 RAT performance is required, especially with regard to evolving mutations. Moreover, cross-reactivity and interference with pathogens and other substances on the test performance of RATs should be consistently investigated to ensure suitability in the context of SARS-CoV-2 containment.

## 1. Introduction

SARS-CoV-2 rapid antigen tests (RATs) are an integral part of SARS-CoV-2 containment strategies. Many RATs have received specific approvals for self-testing and are widely used, for example, in the context of testing strategies in educational institutions or corporate facilities [1,2,3]. In the light of constantly evolving virus variants, it is of particular importance that new SARS-CoV-2 variants of concern (VOCs) do not impair RAT performance. This study was designed as an in vitro approach to investigate if the performance of RATs is affected by VOCs. In addition, a laboratory evaluation was performed to identify potential cross-reactivity with recombinant N-protein of the seasonally circulating human corona viruses (hCoVs) HKU1, OC43, NL63, and 229E. In this study, four common RATs for nasal swab testing were evaluated (Table 1).

## 2. Materials and Methods

### 2.1. Limit of Detection Determination for Cell Culture-Derived SARS-CoV-2

RAT performance was tested with infectious SARS-CoV-2 samples derived from cell culture. Isolation and cultivation of SARS-CoV-2 clinical isolates were performed as described previously [4]. Vero E6 cell culture supernatants containing infectious SARS-CoV-2 were collected 72 h post infection and stored at −80 °C until further use. Infectivity of the used supernatants (50-percent tissue culture infective dose; TCID50/mL) was determined via endpoint dilution assay. All experiments involving infectious SARS-CoV-2 were performed using enhanced biosafety level 3 (BSL-3) containment procedures. The following virus lineages were used: non-VOC B.1.1 and VOCs B.1.1.7 (Alpha), B.1.351 (Beta), P.1 (Gamma), and B.1.617.2 (Delta). Identity of the strains was confirmed by whole-genome sequencing (WGS, for GenBank accession numbers, see paragraph ‘data availability’).

Analytical limits of detection (LoDs) of RATs were determined as described previously [5]. Briefly, dilution series (10-fold dilutions) of infectious virus preparations without heat inactivation were prepared in both Dulbecco’s modified Eagle’s medium (DMEM) and pooled saliva from voluntary SARS-CoV-2 RT-qPCR negative donors. Respective RAT extraction buffers were inoculated with 50 µL of each dilution before following the manufacturers’ instructions for further testing. For each RAT, every dilution was tested in triplicate. The LoD was determined as the dilution level at which two investigators could still identify positive test bands of any intensity in all triplicates blinded with regard to concentration levels. In case of ambiguity, a third investigator was included.

### 2.2. RNA Quantification by RT-qPCR

All dilution levels, including negative controls for both DMEM and saliva, were heat-inactivated for 90 min at 65 °C and tested by RT-qPCR as described previously [5,6]. Briefly, the RNAdvance Viral Large GRP Kit (Beckmann-Coulter Life Sciences, Nyon, Switzerland) was used to extract viral RNA on a Microlab STAR (Hamilton Company, Reno, NV, USA). The RNA was subjected to RT-qPCR with the AmpliCube Coronavirus SARS-CoV-2 RT-qPCR Kit (Mikrogen, Neuried, Germany) on the Bio-Rad CFX96 Real-Time RT-qPCR Detection System (Bio-Rad, Feldkirchen, Germany). For quantification, the EDX SARS-CoV-2 Standard (Exact diagnostics, Fort Worth, TX, USA) was used as a reference RNA standard. 

### 2.3. Limit of Detection for SARS-CoV-2 N-Protein (Non-VOC, B.1.1)

In addition to the infectious virus particles described above, purified, recombinant SARS-CoV-2 N-protein (Mikrogen Diagnostik GmbH, Neuried, Germany) was used for a general comparison of RAT sensitivity. To determine the LoDs of the four RATs, the N-protein was diluted in the respective extraction buffers. Protein amounts from 0.01 ng up to 0.8 ng total inoculum were tested in increments of 0.01 ng (for amounts < 0.1 ng) and increments of 0.1 ng (for amounts from 0.1 to 0.8 ng) to specify the LoD. Testing and LoD interpretation were performed as described above, using 50 µL of the respective dilution and following the manufacturers’ instructions. 

### 2.4. Testing for Cross-Reactivity with Seasonal Human Coronaviruses

To exclude potential cross-reactivity with common seasonal hCoVs, RATs were tested with recombinant N-protein of the hCoV strains HKU1, OC43, NL63, and 229E (Mikrogen Diagnostik GmbH, Neuried, Germany). The N-proteins were diluted in extraction buffers of the respective RATs, and protein amounts of 5 ng and 50 ng total inoculum were tested as described above.

## 3. Results

### 3.1. Rapid Antigen Test Results with SARS-CoV-2 Variants of Concern

The investigated RATs showed an overall comparable LoD with VOCs and the non-VOC B.1.1 (Table 2). In this study, only slight differences between tests from different manufacturers were observed. Generally, all RATs were able to detect all virus variants at least up to a dilution of 1:1000 (corresponding to 2–5 × 10^6^ RNA copies/mL; see Table 3). Additionally, test II displayed positive results at the 1:10,000 dilution (corresponding to 2–6 × 10^5^ RNA copies/mL) for the lineages B.1.1 and B.1.351, independent of the diluent. In contrast, test III detected the lineages B.1.1, B.1.1.7, and B.1.351 at the 1:10,000 dilution only in DMEM, while test IV detected B.1.1 and B.1.351 at the same dilution only in saliva.

### 3.2. Limit of Detection for SARS-CoV-2 N-Protein (Non-VOC, B.1.1)

The LoDs of tests I, II, and IV were 0.3 ng recombinant SARS-CoV-2 N-protein total inoculum, while test III showed a higher sensitivity with an LoD of 0.03 ng total inoculum. (Data not shown.) This result deviated from the VOC testing, where test III showed an overall comparable performance to the other tests.

### 3.3. Testing for Cross-Reactivity with Seasonal Human Coronaviruses

No relevant cross-reactivity was observed using recombinant N-protein from the hCoV strains HKU1, OC43, NL63, and 229E. Only test III showed a weak, non-specific test band in one out of three replicates for both dilutions of hCoV 229E. (Data not shown.)

## 4. Discussion

As new VOCs continue to emerge, it is mandatory that the RATs used for SARS-CoV-2 containment are continuously tested for reliable performance with new VOCs. Mutations in the SARS-CoV-2 genome that lead to alterations of the viral spike (S-) protein affect, e.g., parameters such as transmissibility or immune evasion (cf. N501Y or E484K) [7,8,9]. However, there are also numerous amino acid changes in the nucleocapsid (N-) protein (e.g., aa:N:D3L and aa:N:S235F in B.1.1.7, aa:N:T205I in B.1.351, aa:N:P80R in P.1, aa:N:D63G, aa:N:R203M and aa:N:D377Y in B.1.617.2), which in some cases may alter its dynamic stability and immunogenic properties [8,9,10]. As the majority of currently available SARS-CoV-2 RATs as well as RATs evaluated in this study are based on detection of the N-protein [11], these mutations might influence the test performance of N-specific SARS-CoV-2 RATs, as well as other automated or laboratory-based tests that rely on detection of the N-protein [12,13]. The tests used in our experiments as well as many other antigen detection systems are relying on monoclonal antibodies for the detection of SARS-CoV-2. This strategy has the advantage of high specificity and conformity but might potentially pose difficulties in detecting virus proteins with mutations in the specific target region of those antibodies if only one type of monoclonal antibody is employed.

Previous studies suggest that evaluated RATs were able to detect investigated VOCs, but comprehensive data from independent studies are scarce [5,14,15,16,17]. Our results show that all RATs investigated in this study were able to detect the VOCs B.1.1.7 (Alpha), B.1.351 (Beta), P.1 (Gamma), and B.1.617.2 (Delta) with a comparable performance to the non-VOC B.1.1, although minor variations in the LoD were observed. However, in a clinical setting, pre-analytical factors such as sampling are of enormous importance and highly variable. It is possible that such factors lead to greater variation in test performance than would be the case with minor differences in analytical sensitivity between VOCs. Still, a potential impact of these SARS-CoV-2 VOCs on the performance of RATs with real-life clinical samples cannot be ruled out completely and should therefore be further investigated. Regarding the comparison of analytical sensitivities of the tests, our results indicate very similar LoDs for recombinant N-protein in three out of four tests. These detection limits are generally comparable to those published previously by Corman et al. (2021) [18] with exception of test III, which achieved a considerably lower LoD.

Some limitations of the current study should be noted. The experimental set-up with 10-fold dilution steps limits the resolution of the determined LoDs to the discrete genome copy numbers tested. This may mask differences in LoDs smaller than the tested 10-fold increments, in contrast to the higher LoD resolution with recombinant N-protein. Additionally, slight variations in the initial concentrations of the SARS-CoV-2 virus preparations should be noted (Table 3). Generally, this study should not be considered a clinical validation study but a laboratory-based in vitro study, aiming at analytical performance data. No real-life clinical samples were used. To mimic conditions in biological material, saliva was used as a carrier material in addition to the cell culture medium DMEM. None of the tested RATs is currently approved for use with saliva by their manufacturers. However, no distinct performance differences could be determined between saliva and DMEM. Nevertheless, it must be emphasized that saliva, as a biological material, cannot be standardized, and no conclusions can be drawn from this with regard to clinical applications. Moreover, the test band intensities were quantified by visual inspection instead of software-guided image analysis. This may lead to investigator-dependent variations in the perceived signal strength but, on the other hand, this reflects more accurately the conditions present in the clinical and practical application of those tests.

Another important factor regarding a responsible use of RATs for SARS-CoV-2 containment is the specificity and exclusion of cross-reactivity. The four seasonally circulating hCoV strains HKU1, OC43, NL63, and 229E are particularly closely related to SARS-CoV-2 and cause 15–30% of cases of common cold in human adults [19]. Therefore, cross-reactivity of RATs with these hCoVs should be excluded to avoid frequent false-positive RAT-results [20]. No relevant cross-reactivity with recombinant N-protein of the hCoV strains HKU1, OC43, NL63, and 229E was detected for RATs evaluated in this study, which confirms the manufacturers’ statements and other independent studies [20].

## 5. Conclusions

All RATs evaluated in this study were able to detect the VOCs Alpha, Beta, Gamma, and Delta in a laboratory-based setting with an overall comparable performance to the non-VOC B.1.1. However, these findings pertain to four specific tests under investigation and may not be transferrable to other RATs or VOCs in general. Thus, we suggest that a consistent evaluation of RAT performance regarding potential influences of VOCs and cross-reactivities or interferences should be implemented, to ensure their suitability in the context of SARS-CoV-2 containment. Clinical studies are also urgently needed in this context.

## Figures and Tables

**Table 1:** Rapid antigen tests evaluated in this study.

Test	Name	Manufacturer	Lot #	Expiration Date
**Test I**	SARS-CoV-2 Rapid Antigen Test (self-test) *	Roche, Mannheim, Germany	QCO391041I/Sub I-2	12 January 2023
**Test II**	CLINITEST Rapid COVID-19 Antigen Self-Test *	Siemens Healthineers, Erlangen, Germany	2103544 (a) 2012266 (b)	28 February 2023 (a) 30 November 2022 (b)
**Test III**	Rapid SARS-CoV-2 Antigen Test Card *	Xiamen Boson Biotech Co., Xiamen, China	21032309	September 2022
**Test IV**	Panbio COVID-19 Ag RAPID TEST DEVICE (NASAL)	Abbott, Jena, Germany	41ADG201A SubB (a) 41ADG123A SubA (b)	18 February 2022 (a) 31 January 2022 (b)

* Officially approved for self-testing, according to German approval regulations. # In case of multiple lot numbers, lot (a) was used for VOC testing and lot (b) for further evaluations using recombinant N-protein.

**Table 2:** Limits of detection with cultured strains from non-VOC B.1.1 and VOCs B.1.1.7 (Alpha), B.1.351 (Beta), P.1 (Gamma), and B.1.617.2 (Delta) based on virus dilution series in DMEM and saliva.

Dilution in DMEM						
Test	Virus Lineage	Dilution Levels (RNA copies/mL) ^a^				
		1:10 (10^8^/mL)	1:100 (10^7^/mL)	1:1000 (10^6^/mL)	1:10,000 (10^5^/mL)	1:100,000 (10^4^/mL)
Test I	B.1.1 (Non-VOC)	5	4	2	Neg.	Neg.
	B.1.1.7 (Alpha)	5	3	1	Neg.	Neg.
	B.1.351 (Beta)	5	4	2	Neg.	Neg.
	P.1 (Gamma)	5	3	1	Neg.	Neg.
	B.1.617.2 (Delta)	5	4	2	Neg.	Neg.
Test II	B.1.1 (Non-VOC)	5	4	2	1	Neg.
	B.1.1.7 (Alpha)	5	3	1	Neg.	Neg.
	B.1.351 (Beta)	5	3	2	1	Neg.
	P.1 (Gamma)	5	2	1	Neg.	Neg.
	B.1.617.2 (Delta)	5	3	1	Neg.	Neg.
Test III	B.1.1 (Non-VOC)	5	3	3	1	Neg.
	B.1.1.7 (Alpha)	5	4	2	1	Neg.
	B.1.351 (Beta)	5	4	2	1	Neg.
	P.1 (Gamma)	5	4	2	1 ^b^	Neg.
	B.1.617.2 (Delta)	5	2	1	Neg.	Neg.
Test IV	B.1.1 (Non-VOC)	5	4	2	1 ^b^	Neg.
	B.1.1.7 (Alpha)	5	3	2	Neg.	Neg.
	B.1.351 (Beta)	5	4	2	1 ^b^	Neg.
	P.1 (Gamma)	5	3	1	Neg.	Neg.
	B.1.617.2 (Delta)	5	4	2	Neg.	Neg.
Dilution in Saliva						
Test I	B.1.1 (Non-VOC)	5	4	2	Neg.	Neg.
	B.1.1.7 (Alpha)	5	3	1	Neg.	Neg.
	B.1.351 (Beta)	5	4	2	Neg.	Neg.
	P.1 (Gamma)	5	3	1	Neg.	Neg.
	B.1.617.2 (Delta)	5	4	2	Neg.	Neg.
Test II	B.1.1 (Non-VOC)	5	4	2	1	Neg.
	B.1.1.7 (Alpha)	5	3	2	1 ^b^	Neg.
	B.1.351 (Beta)	5	4	2	1	Neg.
	P.1 (Gamma)	5	3	1	Neg.	Neg.
	B.1.617.2 (Delta)	5	3	1	Neg.	Neg.
Test III	B.1.1 (Non-VOC)	5	3	2	Neg.	Neg.
	B.1.1.7 (Alpha)	5	3	1	Neg.	Neg.
	B.1.351 (Beta)	5	3	2	Neg.	Neg.
	P.1 (Gamma)	5	3	2	Neg.	Neg.
	B.1.617.2 (Delta)	5	2	1	Neg.	Neg.
Test IV	B.1.1 (Non-VOC)	5	4	3	1	Neg.
	B.1.1.7 (Alpha)	5	3	2	Neg.	Neg.
	B.1.351 (Beta)	5	4	2	1	Neg.
	P.1 (Gamma)	5	3	2	Neg.	Neg.
	B.1.617.2 (Delta)	5	4	2	Neg.	Neg.

The test results are described as negative (Neg., no target band visible) or as various degrees of positivity depending on the strength of the test band: (1) very faint, (2) much weaker than control band, (3) weaker than control band, (4) equally strong as control band, and (5) stronger than control band. For LoD determination, all positive bands were considered according to manufacturers’ instruction, irrespective of their intensity. ^a^ Cells shaded in grey colour indicate the dilution determining the LoD. ^b^ Only two of three replicates showed a very faint target band.

**Table 3:** Corresponding RNA copies/mL for all dilution steps based on RT-qPCR.

Dilution in DMEM							
Virus Lineage	Initial Preparation Infectivity (TCID50/mL)	Units	1:10	1:100	1:1000	1:10,000	1:100,000
B.1.1 (Non-VOC)	4.28 × 10^5^	RNA copies/mL	1.9 × 10^8^	2.9 × 10^7^	3.5 × 10^6^	2.7 × 10^5^	1.7 × 10^4^
		Ct value E gene	15.7	18.7	22.0	26.1	30.4
B.1.1.7 (Alpha)	2.85 × 10^5^	RNA copies/mL	2.0 × 10^8^	2.2 × 10^7^	2.9 × 10^6^	3.7 × 10^5^	5.1 × 10^4^
		Ct value E gene	15.7	19.1	22.3	25.6	28.7
B.1.351 (Beta)	2.16 × 10^5^	RNA copies/mL	2.6 × 10^8^	3.1 × 10^7^	3.4 × 10^6^	5.2 × 10^5^	6.9 × 10^4^
		Ct value E gene	15.3	18.6	22.1	25.0	28.2
P.1 (Gamma)	1.77 × 10^5^	RNA copies/mL	2.1 × 10^8^	3.9 × 10^7^	5.3 × 10^6^	5.8 × 10^5^	5.5 × 10^4^
		Ct value E gene	15.6	18.2	21.4	24.8	28.6
B.1.617.2 (Delta)	4.64 × 10^4^	RNA copies/mL	2.8 × 10^8^	3.7 × 10^7^	4.8 × 10^6^	5.4 × 10^5^	6.9 × 10^4^
		Ct value E gene	15.1	18.3	21.5	25.0	28.2
Dilution in Saliva							
B.1.1 (Non-VOC)	4.28 × 10^5^	RNA copies/mL	2.2 × 10^8^	2.4 × 10^7^	2.6 × 10^6^	2.7 × 10^5^	2.7 × 10^4^
		Ct value E gene	15.5	19.0	22.5	26.0	29.7
B.1.1.7 (Alpha)	2.85 × 10^5^	RNA copies/mL	1.6 × 10^8^	1.4 × 10^7^	1.9 × 10^6^	2.3 × 10^5^	2.5 × 10^4^
		Ct value E gene	16.0	19.9	23.0	26.3	29.8
B.1.351 (Beta)	2.16 × 10^5^	RNA copies/mL	1.6 × 10^8^	1.0 × 10^7^	1.8 × 10^6^	≈2 × 10^5^ *	≈2 × 10^4^ *
		Ct value E gene	16.0	20.3	23.1	N.A.	N.A.
P.1 (Gamma)	1.77 × 10^5^	RNA copies/mL	2.1 × 10^8^	2.4 × 10^7^	3.1 × 10^6^	3.9 × 10^5^	4.1 × 10^4^
		Ct value E gene	15.6	19.0	22.2	25.5	29.0
B.1.617.2 (Delta)	4.64 × 10^4^	RNA copies/mL	1.8 × 10^8^	2.1 × 10^7^	2.3 × 10^6^	2.8 × 10^5^	3.8 × 10^4^
		Ct value E gene	15.9	19.3	22.7	26.0	29.1

Ct: cycle threshold; NA: not available; E gene: envelope-protein gene E. * Estimated values based on the 1:1000 dilution step, due to invalid PCR results for the B.1.351 dilution steps 1:10,000 and 1:100,000 in saliva (consistency of the B.1.351 dilution series in saliva was verified in a separate PCR, repeated with the EDX SARS-CoV-2 standard (Exact diagnostics) after 24 h).

## Data Availability

Consensus sequences of the viral lineages determined by WGS were submitted to GenBank with the following accession numbers: B.1.1 (MZ314996), B.1.1.7 (MZ314997), B.1.351 (MZ314998), P.1 (MZ427312), and B.1.617.2 (MZ427313).
